# Distribution and Seasonal Variation of Microplastics in Tallo River, Makassar, Eastern Indonesia

**DOI:** 10.3390/toxics9060129

**Published:** 2021-06-01

**Authors:** Ega Adhi Wicaksono, Shinta Werorilangi, Tamara S. Galloway, Akbar Tahir

**Affiliations:** 1 Department of Fisheries, Universitas Hasanuddin, Jl. Perintis Kemerdekaan, KM 10 Tamalanrea, Makassar 90245, Indonesia; egaadhi@gmail.com; 2 Department of Marine Science, Universitas Hasanuddin, Jl. Perintis Kemerdekaan, KM 10 Tamalanrea, Makassar 90245, Indonesia; shintakristanto@yahoo.com; 3 College of Life and Environmental Sciences: Biosciences, University of Exeter, Geoffrey Pope Building, Stocker Road, Exeter EX4 4QD, UK; T.S.Galloway@exeter.ac.uk

**Keywords:** plastics, riverine, coastal, estuary, characteristics, pollution

## Abstract

Attention towards microplastic (MP) pollution in various environments is increasing, but relatively little attention has been given to the freshwater-riverine environment. As the biggest city in the eastern Indonesia region, Makassar can be a potential source of MP pollution to its riverine area. This study aimed to determine the spatial trends, seasonal variation, and characteristics of MPs in the water and sediment of Tallo River, as the main river in Makassar. Water samples were collected using a neuston net and sediment samples were collected using a sediment corer. The samples collected contained MPs with an abundance ranging from 0.74 ± 0.46 to 3.41 ± 0.13 item/m^3^ and 16.67 ± 20.82 to 150 ± 36.06 item/kg for water and sediment samples, respectively. The microplastic abundance in the Tallo River was higher in the dry season and tended to increase towards the lower river segment. Fragments (47.80–86.03%) and lines (12.50–47.80%) were the predominant shapes, while blue (19.49–46.15%) and transparent (14.29–38.14%) were the most dominant color. Polyethylene and polypropylene were the common MP polymers found in the Tallo river. Actions to prevent MP pollution in the Makassar riverine area are needed before MP pollution becomes more severe in the future.

## 1. Introduction

Plastic pollution is being reported everywhere and has become a major global problem. An increasing amount of plastic waste, primarily caused by anthropogenic activities in terrestrial locations, may eventually end up in the sea [1,2]. More than 190 coastal countries have been identified as contributors to an annual release of up to 12.7 million metric tons of plastic debris into the ocean [2]. Environmental stressors such as physical abrasion, elevated temperature, and UV-B exposure can all help plastic waste degrade into a smaller form of plastic in the environment [3,4]. These small-sized plastic particles that range from 1–5 mm eventually merge into a new form, called “microplastic” [5,6].

Microplastics (MPs) tend to receive a lot of attention from researchers, public communities, and governments worldwide due to their potential impacts on the ecosystem [7,8,9]. Microplastics are known to interact with other toxic compounds in the aquatic ecosystem [10,11,12]. Internal compounds in the MPs may also induce toxicity to the exposed organism [13]. The shape of MPs can resemble plankton, the primary food source in the aquatic environment, which makes it very easy to be consumed by aquatic organisms [14,15]. Reports on the incidence of MP ingestion by aquatic organisms have also been widely reported, as in plankton, fish, and shellfish [16,17,18,19,20]. This situation raises concerns about MPs’ impact not only on the ecosystem but also on food security, which may have implications for human health [21,22].

Indonesia is branded as the world’s second-largest contributor to ocean plastic pollution [2]. However, research regarding MP pollution in Indonesia is still in its early stages and needs further development. Currently, research on MP pollution in Indonesia focuses more on the marine environment. Microplastic is known to contaminate sediment [23,24], water [25,26] and biota [16,27,28] in Indonesia’s marine environment. In contrast, research on MPs in the freshwater environment in Indonesia has received little attention. Only a few studies concerning MP pollution have been conducted in Indonesia’s rivers [29,30]. According to these studies, MPs are reported to pollute rivers in the western Indonesia region, especially on Java Island [31].

To the best of our knowledge, even though studies regarding MPs have been conducted in western Indonesia’s river, no MP pollution research has ever been performed in the riverine area in Indonesia’s eastern region. Eastern Indonesia is an important location for plastic pollution research. This area is passed by the Indonesian throughflow (ITF) ocean current, which can carry plastic waste from the pacific ocean and its stream trajectory to the Indian Ocean [32,33]. The high input of plastic debris from the rivers in eastern Indonesia due to ITF ocean currents can further spread to other locations, posing risks to broader geographical areas.

As the biggest city in eastern Indonesia, Makassar needs more attention due to high anthropogenic pressure. Shuker and Cadman, in 2018 [34], reported that Makassar City produces more than 1200 tons of solid waste a day. The same report also stated that more than 44% of trash found in the Makassar coastal area is plastic waste. The coastal area of Makassar is already polluted by plastic waste in several colors and sizes [35,36]. The estuary areas in Makassar City also show MP contamination suspected from the river outflow [37]. Despite research into MPs in the marine environment of Makassar City being conducted at least five years earlier [16,33,35,38], information regarding MP pollution in Makassar’s riverine environment is still lacking.

This study focuses on the MP pollution in Tallo River, as the main river trajectory in Makassar City. In general, Tallo riverbank is still covered by a mangrove ecosystem, as this river is utilized for recreational and fisheries purposes. The occurrence of MPs in Tallo River may pose threats to human health in Makassar City, considering that most of the freshwater fish and shrimp commodities in Makassar originate from this river. Tallo River is also directly feeding the Makassar Strait, the location of the ITF ocean current. This research aims to determine the abundance, spatial trend, and characteristics of MPs in the water and sediment of Tallo River during the wet and dry seasons. This research provides novel data on MP pollution in Makassar’s riverine environment. It could be used as a baseline to evaluate and improve solid waste management in the east Indonesia region, particularly in Makassar City.

## 2. Materials and Methods

### 2.1. Study Sites and Sampling

**Figure 1:**
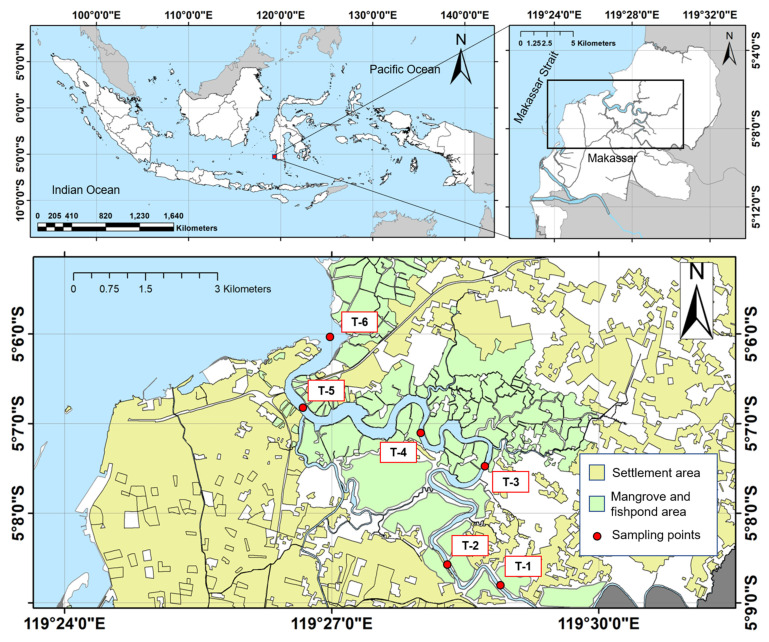
Sampling points on Tallo River.

The study was conducted in the section of the Tallo River that crosses Makassar City, Indonesia. Samples were taken in March and August 2019 to represent the wet and dry seasons, respectively. Six sampling points were distributed purposively based on their position from the upstream to the downstream part of the river section. Sampling points 1 and 2 were located on the upstream part of the river, where there is a thick *Nypa fruticans* green belt on the riverbank in this river segment. The mid-stream section was represented by sampling points 3 and 4, which are surrounded by a mangrove ecosystem and fisheries activities, such as a fish and shrimp pond. Between points 3 and 4, a flow of water enters from the Makassar industrial area. The downstream segment was represented by sampling points 5 and 6, which are surrounded by Makassar City’s slum district. There is also a water flow that enters the Tallo river at point 5, originating from the Makassar urban area. Land use/cover area [39] and sampling points on Tallo River are described in Figure 1.

Water samples were collected in triplicate from each sampling point using the neuston net method [26] with a slight modification to the net dimension. A custom rectangle-mouth neuston net (15 × 60 cm, 330 µm mesh size) was towed perpendicular to the river current at a constant speed (4 km/h) using a boat. Towing distance was measured using a GPS device (Garmin Montana 680, Schaffhausen, Switzerland). The amount of water filtered during towing was calculated by multiplying the net mouth area with the towing length. Water accrued in the cod-end was then transferred into a bottle sample and added to 30 mL of 10% KOH solution [40]. Following that, the samples were transported to the laboratory in a cool box. Water samples were preserved at 4 °C prior to further analysis. Samples were then filtered using a vacuum pump (Rocker 410, Kaohsiung, Taiwan) to a sterile 0.45 µm pore size cellulose filter (Whatman GE 7141-104, Buckinghamshire, UK). The filter paper was then placed into a clean glass Petri dish to be observed visually using a stereomicroscope.

Bulk sediment samples were taken in triplicate at every sampling point using a sediment corer (Ø 4.9 cm) in the river littoral zone (50 cm–1 m depth) [14]. Sediment was collected from the riverbed’s top layer (5–7 cm). Sediment samples were then transferred to a Ziplock bag and preserved in the cool box for further analysis in the laboratory.

Sediment samples (400 g wet weight) were dried in an oven (60 °C for 48 h). For the density separator process, a total of 100 g of dry weight (DW) sediment was taken from the dried samples and subjected to 300 mL of a 30% NaCl solution (337 g analytical NaCl powder + 1 L distilled water, density ≈ 1.2 g/cm^3^) [41]. The samples were stirred at 1200 rpm for 2 min using a magnetic stirrer. Sediment samples were left at room temperature (27–28 °C) overnight to create a supernatant layer in the sample. The supernatant liquid was then filtered using the same method as that used in the water samples procedure described. The filter paper was then placed in a clean glass Petri dish for further visual analysis using a stereomicroscope.

Visual observations were performed using a stereomicroscope (Euromax SB-1902, Arnhem, Netherland; 45× magnification). The filter paper inspection was performed using a zigzag movement on filter paper until all of the areas on the filter were observed. Any MPs found in the filter paper were taken and placed into an object glass for preservation. The number, shape, size, and color of the MPs were then determined. The MPs’ colors were classified according to Frias et al. [42] and the MPs’ shape identification referred to GESAMP [43]. The MPs’ size was determined using ImageJ (National Institute of Health, Bethesda, MD, USA, version 1.52a) software. Microplastic sizes were then classified into small MPs (SMPs, <1 mm) and large MPs (LMPs, 1–5 mm) [29,44]. The abundance of MPs in the samples was expressed in items/m^3^ for water and items/kg DW for the sediment samples.

The polymer types of the representative MP samples were identified separately using the Fourier-transform infrared spectroscopy (FTIR) method. Microplastic was placed in the sample chamber and read using the FTIR machine (Bruker Tensor II, Ettlingen, Germany) with ATR accessories in a 500–4000 cm^−1^ spectral range and resolution of 4 cm^−1^. The wave spectrum was then matched with the NICODOM spectra library to determine the polymer type.

### 2.2. Quality Controls

Several actions were taken to prevent contamination in the samples. All of the pieces of equipment were pre-cleaned with tap water and rinsed with distilled water. The MPs visual observation workspace was also cleaned using a dust roller prior to the MP identification process. All of the filter-filled Petri dishes were kept closed to prevent airborne contamination. During the visual observation process, Petri dish covers were opened for no longer than 30 s for every MP found, in order to move the MPs from the filter paper to an object glass.

Sample blanks and airborne controls were used as the negative control. A total of 12 sediment and 12 water sample blanks were created during this research. Water sample blanks were created by rinsing the clean neuston net from the net mouth with distilled water before towing. The flushed distilled water in the net cod-end was kept and analyzed as other water samples. For the sediment sample blanks, about 600 mL of the NaCl solution used in the density separator was filtered before use. The filter was then observed using the stereomicroscope.

Airborne controls were performed by placing three opened Petri dishes filled with distilled water next to the microscope during the visual observation process. Controls were placed 10 min before the sample observation and taken 10 min after the MPs visual analysis was complete. Controls were then observed visually using the same method that was used for the samples.

### 2.3. Data Analysis

The trends in MP abundance in water and sediment were analyzed using a one-way ANOVA with Tukey’s post hoc analysis to determine the spatial MP abundance between the sampling points. The significant difference in MP abundance between the wet and dry seasons was determined using a parametric *t*-test. Microplastic color, shape, size and polymers were presented descriptively. Spatial distribution graphics and statistical analysis were conducted using GraphPad Prism (Graphpad Software, San Diego, CA, USA, version 9.0.2).

## 3. Results and Discussions

### 3.1. Contamination Control

Microplastic was not found in all water and sediment sample blanks. In the negative airborne control, from the 45 Petri dishes used during the MP identification process, only 1 MP (line, purple) was found with the average MP abundance found to be 0.02 items/Petri dish. Microplastic in the airborne blanks only had a proportion of about 0.28% of the MPs found in samples. Therefore, it is assumed that contamination does not affect the MPs’ identification in water and sediment samples and can be ignored.

### 3.2. Microplastic Abundance on Water and Sediment

A total of 36 water and 36 sediment samples from the Tallo River were analyzed in this research. Microplastic was found in all of the samples. Microplastics are widespread in various environments, including the riverine system [14,45]. Mostly, the MPs found in the freshwater system come from anthropogenic pressures such as domestic, industry, wastewater treatment plants, and agrosystems [46,47]. All of the samples observed in this study contained MPs, which indicates that MPs have contaminated Tallo River.

**Figure 2:**
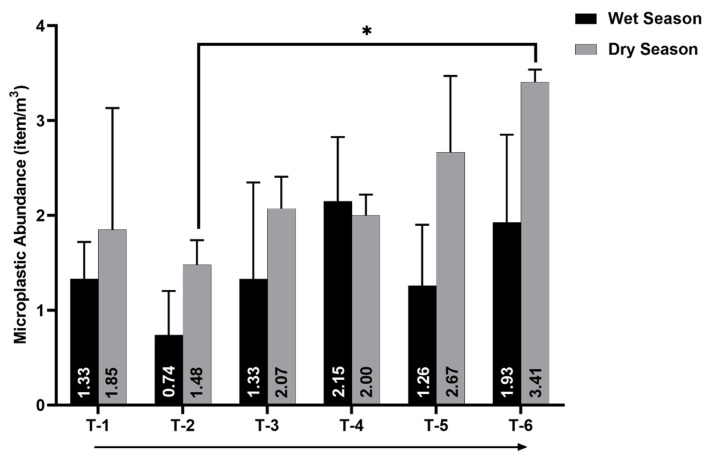
Microplastic abundance on the surface water of Tallo River. The arrows below the graph indicate the position of sampling points from the upstream to the downstream part of the river. The error bar indicates standard deviation (*n* = 3). The asterisk indicates the significant difference between sites based on a one-way ANOVA (*p* < 0.05).

The microplastic abundance found in water samples ranged from (mean ± SD) 0.74 ± 0.46 to 2.15 ± 0.68 items/m^3^ in the wet season and 1.48 ± 0.26 to 3.41 ± 0.13 items/m^3^ in the dry season (Figure 2). The microplastic abundance in water samples in this study is considered much lower than that which was reported in other river locations in Indonesia. Ciwalengke and Surabaya River in Indonesia were reported to have a MP abundance up to 600 items/m^3^ and 21 items/m^3^, respectively [29,30]. This result is understandable because the Ciwalengke and Surabaya Rivers flow directly through a densely populated district and an industrial area, which provide potential sources of MP pollution. In contrast, the Tallo River is mainly covered by mangrove areas on its riverbank and is not directly bordered by a resident/industrial area. The existence of mangrove areas could act as a MP trap. The muddy mangrove sediment could trap MPs and increase the magnitude of MP abundance up to eight times compared to non-mangrove sediment [48]. A mangrove ecosystem in the Tallo riverbank might prevent the run-off leakage of MPs entering the river. This condition could contribute to the lower MP abundance in the river water.

**Figure 3:**
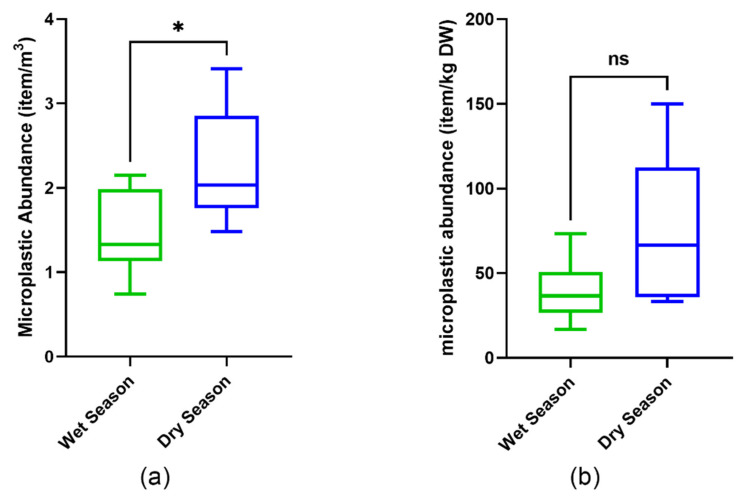
Boxplot diagram of microplastic abundance in water (**a**) and sediment (**b**) during the wet and dry seasons in Tallo River. The asterisk indicates the significant difference between the sites based on a *t*-test (*p* < 0.05). ns indicate no statistical difference between the sites based on a *t*-test (*p* > 0.05) explanation.

Microplastic abundance in water samples was significantly higher in the dry season (2.247 ± 0.688 items/m^3^) compared to the wet season (1.457 ± 0.508 items/m^3^) (*p* < 0.05) (Figure 3). In comparison, there was no significant difference in MP abundance in the sediment samples between the two seasons (*p* > 0.05). The tendency for a higher concentration of MP abundance in the dry season also happens in other rivers, such as the Maozhou and Yellow Rivers in China [49,50]. The difference in MP abundance in riverine water could happen because of the variation in topography, precipitation, and waste management in the sampling locations [49]. The Tallo River itself has a wide variety of water depths and velocities between the wet and dry seasons. Water depth in Tallo River during the wet season is due to high precipitation, and can be two times deeper than the depth during the dry season [51]. This difference could cause the river water volume:surface-water area ratio to be smaller in the dry season, which leads to a higher amount of MPs in the surface water [18].

**Figure 4:**
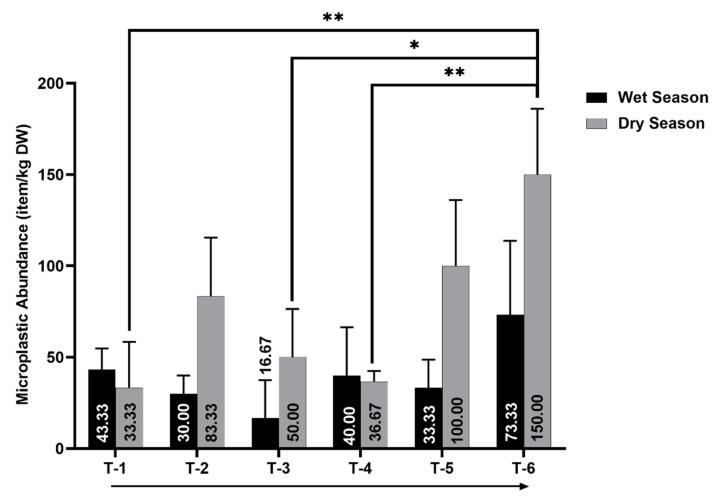
Microplastic abundance in sediment from Tallo River. The arrows below the graph indicate the position of every site from the upstream to the downstream part of the river. The error bar indicates standard deviation (*n* = 3). The asterisk (*) indicates the significant difference between the sites based on a one-way ANOVA (*p* < 0.05). The double asterisks (**) indicate the higher significant difference between the sites (*p* < 0.01).

The microplastic abundance in sediment samples from Tallo River varied from 16.67 ± 20.82 to 73.33 ± 40.41 items/kg DW in the wet season and 33.33 ± 25.17 to 150 ± 36.06 items/kg DW in the dry season (Figure 4). The microplastic abundance in sediments from Tallo River was also considered lower compared to the other river sediments in Indonesia, such as in Ciwalengke River (≈300 items/kg DW), Jagir Estuary (90 to 590 items/kg DW), and Estuary in Jakarta Bay (up to 38,000 items/kg DW) [24,30,52]. This result suggests that MP abundance in Tallo sediment might not be as severe as that reported in riverine sediments from Java Island, the most populated island in western Indonesia. The higher anthropogenic pressures on the river catchment area will mostly lead to a higher MP abundance in its river environment. Jakarta City, where the MP abundance in riverine sediment exceeded 15,000 items/kg DW, for example, has a population of more than 10 million people [53], about 7.5 times higher than the population of Makassar City.

The microplastic in water and sediment from the Tallo River has a similar spatial distribution. The microplastic abundance in the Tallo River tends to be higher in the river-mouth area compared to the upstream area. This pattern was more observable in the dry season. The microplastic abundance at site T-2 was significantly lower compared to site T-6, which was located at the river-mouth during the dry season (*p* < 0.05) (Figure 2). In the sediment samples, sites T-1, T-3, and T-4 were significantly lower compared to site T-6 (*p* < 0.05) (Figure 4). Even though there was no statistical difference in MPs’ spatial distribution during the wet season, a similar trend to the dry season was observed, where the Tallo River’s downstream segment had a greater MP abundance compared to the upstream section. An estuary location is more susceptible to MP contamination. The Tallo Estuary riverbank is directly located next to the slum settlement area of Makassar City, which potentially gives MPs input to the Tallo downstream area. Settlement area can provide various MP sources (e.g., laundry waste, beads from personal care products, and domestic trash) [14,54,55]. Estuaries with high anthropogenic pressure will generally have a higher MP abundance [47]. Water velocity in the estuary, in general, is lower than in the upstream river due to the more static marine water mass that influences this area. MPs’ transport in the river is strongly affected by flow regime. The intense flow can cause the MPs’ mobilization and transport, while the low stream velocity is causing the MP retention and deposition [56,57]. Low water velocity in Tallo Estuary can lengthen the MPs’ residence time, leading to MPs’ accumulation and increment in the estuary area.

### 3.3. Microplastic Characteristics

#### 3.3.1. Microplastic Color

**Figure 5:**
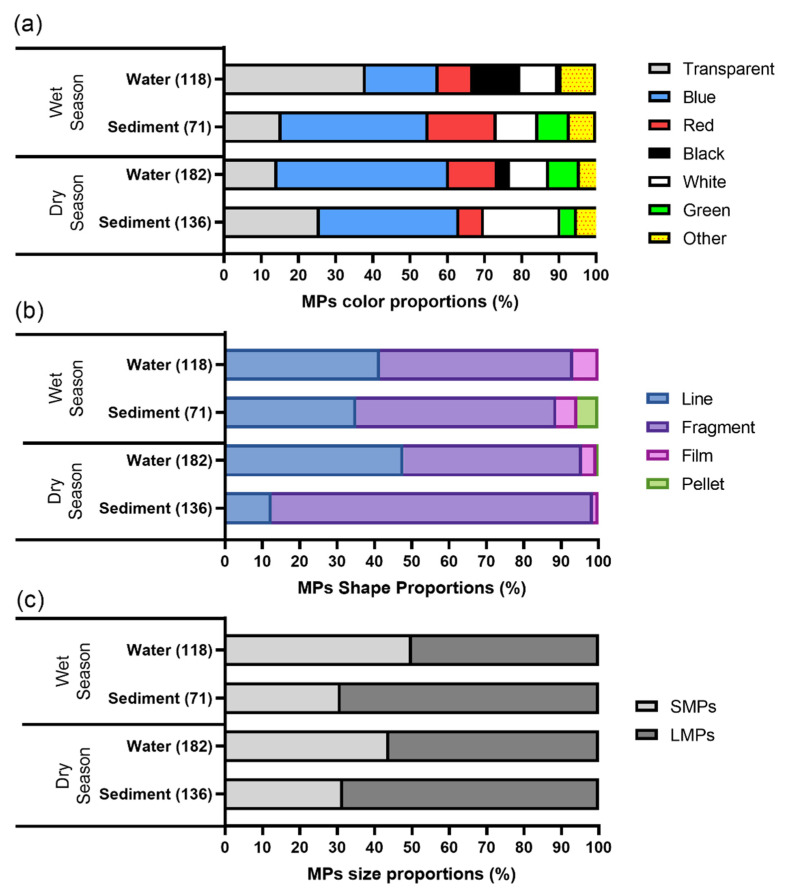
The proportions of the MPs’ colors (**a**), shapes (**b**) and sizes (**c**) in the water and sediment samples from Tallo River. SMPs, small microplastics (<1 mm); LMPs, large microplastics (1–5 mm).

In general, there were six prominent MP colors found in the samples (Figure 5). Blue (19.49–46.15%) and transparent (14.29–38.14%) were the most dominant MP colors found in Tallo River, followed by white (10.17–20.59%), red (6.62–18.31%) and green (0.85–8.45%). Black MPs in Tallo River were only found in the water (3.30–12.71%) and were not present in the sediment compartment.

Microplastic color can provide information to predict the source and weathering process of MPs. For example, transparent color is often associated with polypropylene, commonly used as a food packaging material. The yellowish color of MPs can also indicate the photooxidation and weathering process of MPs [58]. In Tallo River, the most common MP colors found were blue and transparent. The pigmented MPs color may originate from textile and paint, which usually use various colors. The transparent MPs can be linked to a transparent food container that mainly consists of polyethylene and polypropylene polymer. The color of MPs may also influence a fish’s preference to eat small plastic particles. Fish tend to prefer MPs with a similar color to their prey. For example, the scad fish collected from the South Pacific Gyre tend to ingest blue MPs due to their color similarity to the copepod species, which is scad’s natural prey [59]. Some authors report that fish tend to prefer lighter colors of MPs, such as blue, white, and transparent, because it is easier to distinguish these colors compared to the brownish natural environment color [37,60]. The dominance of blue and transparent MPs might make these MPs more bioavailable for the aquatic organism in the river. In addition, a MPs’ color usually comes from a synthetic colorant that can leach into the environment and pose additional risks to the aquatic organism [13].

#### 3.3.2. Microplastic Shape

**Figure 6:**
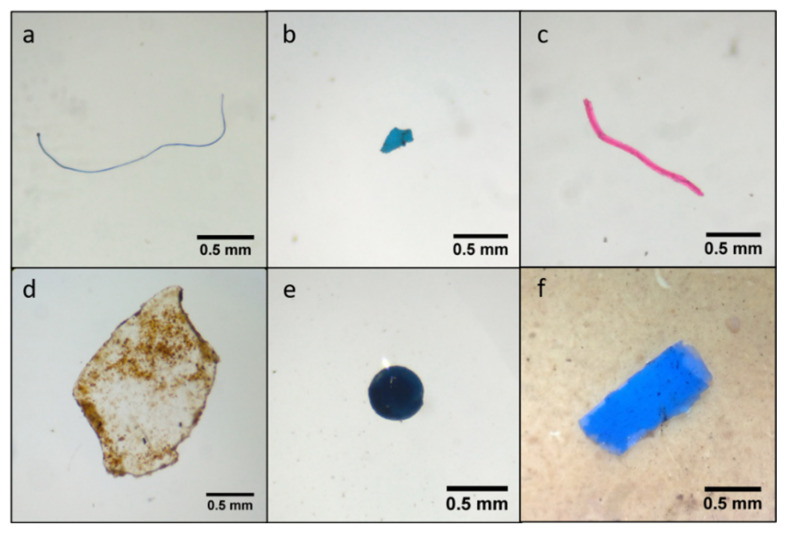
Representative of MPs found in the samples. Blue and red line (**a**,**c**), blue fragment (**b**), transparent fragment (**d**), blue pellet (**e**), and blue film (**f**) MPs.

The microplastics in Tallo River were dominated by fragments (47.80–86.03%) and lines (12.50–47.80%) compared to other MP shapes, such as films (1.47–6.78) and pellets (0.55–5.63%) (Figure 5 and Figure 6). A higher pellet proportion existed in Tallo sediment during the dry season (5.63%), while in the wet season, it only had a proportion of about 0.5% in the water. Tallo sediment during the dry season had a significant proportion of fragments.

The shape of MPs could mimic the natural prey of fish that exist in the environment [14,15]. For example, the line type of MPs has a similar shape to the filamentous algae in the aquatic environment, which is a fish’s natural prey. The MPs’ shape can also be an indicator of the MPs’ origins. Fragments mainly originate from a secondary source of MPs (fragmentation of larger-sized plastic) [61]. The existence of pellets also shows the probability of primary MPs. Tallo River also receives water flow from the Makassar Industrial Area, where several plastics industries might be using the preproduction plastic pellet. Plastic pellets can leak into the environment due to production processes and raw pellet transportation [55]. However, the low proportion of pellets in this study suggests that MPs in the Tallo River do not primarily originate from primary MPs.

#### 3.3.3. Microplastic Size

In general, there are a higher proportion of LMPs (50–69.01%) in the Tallo Riverine environment than SMPs (30.99–50%). Microplastic found in the water tends to be smaller compared to MP found in the sediment compartment. A more significant proportion of LMPs in the Tallo River suggests that the MPs have not been further degraded. In a long trajectory river such as the Rhine River in Europe, SMP tend to dominate [62]. A large proportion of SMPs can indicate further plastic degradation due to physical and chemical stressors from the environment. The size of MPs can be gradually reduced because of degradation mechanisms in the river’s trajectory. As MPs move towards river mouths, they can degrade to a smaller size. This condition leads to a higher proportion of SMPs in lower river segments [52].

Moreover, MPs’ dimensions also affect their possible bioavailability. Microplastics with smaller sizes can be more easily ingested by zooplankton, making it easier for SMPs to enter the food web [20]. It is also easier for small-sized MPs to be transported into an organism’s soft tissue, posing a greater risk to the organism [63].

#### 3.3.4. Microplastic Polymer

**Figure 7:**
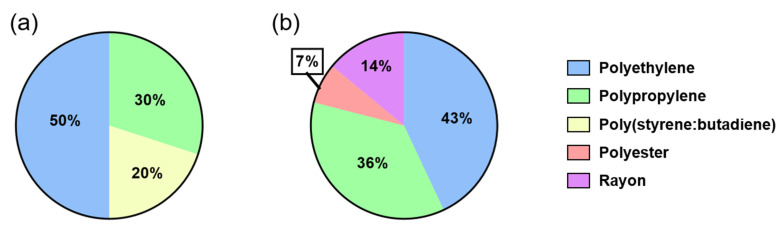
Microplastic polymer identified in water (**a**) and sediment (**b**) samples.

A total of five polymers were identified in the study site (Figure 7). The most predominant polymers found in the water and sediment samples were polyethylene (43–50%) and polypropylene (30–36%). Poly(styrene:butadiene) was only found in the water samples (20%). Synthetic rayon and polyester were only found in the sediment samples (14% and 7%, respectively). Poly(styrene:butadiene) and polyethylene were mainly found in the shape of fragments, while polypropylene, rayon and polyester were found in the form of lines. As the highest-produced polymer globally, polypropylene and polyethylene are more available to reach the aquatic environment [64]. This condition means that polyethylene and polypropylene are commonly found in freshwater environments [65]. Poly(styrene:butadiene) is mainly used for anti-abrasion surfaces, such as in car tires and shoe soles, while rayon and polyester are commonly used as textile material [43,54,66,67]. A single wash of about 6 kg of polyester clothes can release nearly 500,000 polyester fibers in its waste effluent, leading to a higher polyester line in the environment [54]. The low density of polystyrene-butadiene (0.94 g/cm^3^) means this polymer commonly accumulates in surface water. In contrast, rayon and polyester have a higher density than 1.35 g/cm^3^, higher than the water density [43]. This condition means rayon and polyester tend to sink in the environment and end up in the sediment compartment.

## 4. Conclusions

Tallo River has been contaminated by MPs, both in the water and sediment compartment. The MP abundance in the Tallo River is influenced by seasonal variations, where the MP abundance is higher in the dry season. The spatial trends suggest that MP abundance in the Tallo River tends to be higher in the lower river segment. Microplastics in the Tallo river mainly originate from secondary MPs, and polyethylene and polypropylene in the form of lines and fragments dominate. This is the first report of MP pollution in eastern Indonesia’s river. The low MP abundance in water and sediment compared to that which is reported on the highly populated Java Island should be an incentive for early action to prevent MP contamination in Tallo River becoming more severe in the future.

## Data Availability

Research data are available on request from the corresponding author.
